# Accuracy of the Cosmed K5 portable calorimeter

**DOI:** 10.1371/journal.pone.0226290

**Published:** 2019-12-16

**Authors:** Scott E. Crouter, Samuel R. LaMunion, Paul R. Hibbing, Andrew S. Kaplan, David R. Bassett

**Affiliations:** Department of Kinesiology, Recreation, and Sport Studies, The University of Tennessee Knoxville, Knoxville, TN, United States of America; University of Mississippi, UNITED STATES

## Abstract

**Purpose:**

The purpose of this study was to assess the accuracy of the Cosmed K5 portable metabolic system dynamic mixing chamber (MC) and breath-by-breath (BxB) modes against the criterion Douglas bag (DB) method.

**Methods:**

Fifteen participants (mean age±SD, 30.6±7.4 yrs) had their metabolic variables measured at rest and during cycling at 50, 100, 150, 200, and 250W. During each stage, participants were connected to the first respiratory gas collection method (randomized) for the first four minutes to reach steady state, followed by 3-min (or 5-min for DB) collection periods for the resting condition, and 2-min collection periods for all cycling intensities. Collection periods for the second and third methods were preceded by a washout of 1–3 min. Repeated measures ANOVAs were used to compare metabolic variables measured by each method, for seated rest and each cycling work rate.

**Results:**

For ventilation (V_E_) and oxygen uptake (VO_2_), the K5 MC and BxB modes were within 2.1 l/min (V_E_) and 0.08 l/min (VO_2_) of the DB (p≥0.05). Compared to DB values, carbon dioxide production (VCO_2_) was significantly underestimated by the K5 BxB mode at work rates ≥150W by 0.12–0.31 l/min (p<0.05). K5 MC and BxB respiratory exchange ratio values were significantly lower than DB at cycling work rates ≥100W by 0.03–0.08 (p<0.05).

**Conclusion:**

Compared to the DB method, the K5 MC and BxB modes are acceptable for measuring V_E_ and VO_2_ across a wide range of cycling intensities. Both K5 modes provided comparable values to each other.

## Introduction

In recent years, portable metabolic measurement systems have been developed that are worn on the body allowing for measurements of energy expenditure to be done in the field (e.g. free-living environments), over extended time periods. For comprehensive reviews on portable indirect calorimeter systems see reviews by Overstreet et al. [1] and Macfarlane [2]. In general, portable systems are used for a number of applications, including: 1) measuring maximal oxygen uptake (VO_2max_) in sport-specific settings, 2) quantifying the energy cost of physical activities in free-living environments, and 3) calibrating and validating accelerometer-based wearable devices that assess physical activity in laboratory and free-living environments (up to 6 continuous hours of measurement).

Cosmed, L.L.C. (Rome, Italy) recently introduced a new portable indirect calorimeter system called the Cosmed K5 (see methods for full specifications), to replace the Cosmed K4b^2^. The K4b^2^ uses the breath-by-breath (BxB) technique for measurement of respiratory gas exchange and has been shown to have mean errors of <96 ml/min for oxygen uptake (VO_2_) measurements, compared to the Douglas bag (DB) technique during rest and stationary cycling between 50 and 250 W [3]. Carbon dioxide production (VCO_2_) and ventilation (V_E_) values from the K4b^2^ were lower than DB values at 200–250 W, but there were no significant differences for rest through 150 W. Several other studies have examined the validity of the K4b^2^ with similar results [4–6]. In general, the differences between the K4b^2^ and DB are not considered to be of practical significance (group level error < 5%), thus the K4b^2^ is viewed as having acceptable accuracy for most applications [7, 8].

The new K5 is capable of measuring respiratory gas exchange by the BxB technique, similar to its predecessor (K4b^2^). However, the K5 now has the ability to measure respiratory gas exchange through the use of a dynamic mixing chamber (MC) that uses a constant flow pump, which is useful for assessing steady-state metabolic rates. With the MC, expired gas samples from multiple breaths are collected and stable F_E_O_2_ (fraction of expired oxygen) and F_E_CO_2_ (fraction of expired carbon dioxide) values are obtained. Recently, Guidettie et al. [9] performed a systematic evaluation comparing the K5 BxB mode against a metabolic simulator. Overall, there were no significant differences in mean values between the K5 and simulator, for V_E_ (-0.50%, p = 0.11), VO_2_ (-0.04%, p = 0.80), or VCO_2_ (1.03%, p = 0.09). Intra- and inter-device reliability of the two K5 units tested was high (Intra-class correlations (ICCs) > 0.99; mean absolute percent error (MAPE) < 2%), with no significant difference between trials [9]. Perez-Suarez et al. [10] compared the K5 MC and BxB modes to the Vyntus CareFusion stationary metabolic cart during rest and cycling at 60W and 130-160W. For rest and both cycling intensities, the K5 MC and BxB mode were within 13.4% of VO_2_ measured by the Vyntus. In general, the K5 BxB mode was closer to the Vyntus VO_2_, VCO_2_, and respiratory exchange ratio (RER) at rest and 60W cycling. At the highest cycling intensity, the K5 BxB mode was approximately 6.6% lower than the Vyntus VO_2_ while the K5 MC mode was 5.8% higher than the Vyntus VO_2_.

To date, the Cosmed K5 BxB and MC modes have not been validated against the traditional criterion method (i.e., the DB method) in humans, at rest and over a wide range of cycling intensities. Thus, the purpose of this study was to compare respiratory gas exchange variables from the K5 BxB and MC modes to DB (criterion method) in healthy adults, during seated rest and cycle ergometry at fixed work rates between 50 and 250 W. Additionally, respiratory gas exchange variables were compared between the K5 BxB and MC modes.

## Materials and methods

### Participants

Fifteen healthy participants (14 males) from the Knoxville, TN community volunteered to participate in the study. Due to the length of the cycling protocol and the prescribed work rates, we used a convenience sample of trained cyclists. Specifically, we recruited individuals who could cycle continuously for 90 minutes and had the ability to cycle at 250 W for at least 15 minutes. The procedures were reviewed and approved by The University of Tennessee Knoxville Institutional Review Board, before the start of the study. Each participant signed a written informed consent and completed a health history questionnaire before participating in the study. Participants were excluded from the study if they had any contraindications to exercise.

### Equipment

The Cosmed K5 is a portable metabolic system that is worn on the back with a harness and the unit measures 174x111x64 mm and weighs 900g (including battery and oxygen (O_2_) sensor). The K5 has a 3.5 in LCD display, is capable of USB and Bluetooth PC communication, has a rechargeable Li-Ion “Smart battery” with LCD charge status that lasts up to 4 hours, and has a storage capacity for up to 2,048,000 breaths. Additional features also include IP54 standard (rugged design, weather sealed, waterproof and dust-proof), a user-replaceable O_2_ sensor, SD-card slot for extra storage capacity, tripod mount, a 10Hz GPS/QZSS receiver, altimeter (using barometric pressure + GPS offset), ANT+ capability, and an updated OMNIA Metabolic software. The standard K5 uses a micro-dynamic MC for measurement of VO_2_ and VCO_2_ and there is an option for a dual system that also has the capability to perform BxB measurements. For the current study we validated the dual mode system. The K5 uses a galvanic fuel cell for the O_2_ analyzer (response time, 120 ms; range, 0–100%), a digital infrared carbon dioxide (CO_2_) analyzer (response time, 100 ms; range, 0–10%), and proprietary software (Firmware v1.3 01252018 used in the current study). The flowmeter uses a bi-directional digital turbine that has a flow range of 0.08–16 l/s. The flow meter is connected to a flexible Hans-Rudolph V2 facemask with inspiratory valves that covers the participant’s mouth and nose. A Permapure sampling line dries the gas sample collected at the facemask prior to being analyzed by the gas analyzers. For this study, the same dual mode K5 was used for all testing and prior to all tests, the K5 was calibrated according to the manufacturer’s instructions. This consists of: 1) a room air calibration, 2) a flow meter calibration using a 3-L syringe, 3) a scrubber calibration that zeros the CO_2_ analyzer, 4) reference gas calibration using a known reference gas (16% O_2_, 5% CO_2_, 79% nitrogen (N_2_)); this was done separately for the MC and BxB modes, and 5) a delay calibration for the BxB mode.

DB collections of expired gases were made using a mouthpiece connected to a 2-way Hans-Rudolph breathing valve (2700 series) and a 2-meter corrugated hose. At the end of each DB collection period, the gas fractions (F_E_O_2_ and F_E_CO_2_) from the DB were measured (over a 1-minute sampling period) using a paramagnetic O_2_ analyzer (response time, 200 ms; range, 0–25%) and an infrared, single beam, single wave-length CO_2_ analyzer (response time, 100 ms; range, 0–10%). A Permapure sampling line was connected between the DB and gas analyzers to dry the gas. Prior to each test, the gas analyzers were calibrated using room air and a known reference gas (15.09% O_2_, 4.01% CO_2_, 80.9% N_2_). After the gas samples were measured, the expired volume was determined by pushing the remaining collected expired gas from the DB into a 120-L Tissot gasometer (Warren E. Collins, Braintree, MA). Corrections were made for the volume of air removed for gas analysis to obtain the total expired volume. BTPS (body temperature pressure saturated) and STPD (standard temperature pressure saturated) were calculated for each measurement using the barometric pressure, ambient pressure, and vapor pressure using the following formulas:

$$V\;(BTPS)=V\;(ATPS)\frac{Barometric\;pressure-Water\;vapor\;pressure}{Barometric\;Pressure-47\;mmHg}\times \frac{273{}^{\circ}\;K+37{}^{\circ}\;C}{273{}^{\circ}\;K+Ambient\;temperature}$$

$$V\;(STPD)=V\;(ATPS)\frac{Barometric\;pressure-Water\;vapor\;pressure}{760\;mmHg}\times \frac{273{}^{\circ}\;K}{273{}^{\circ}\;K+Ambient\;temperature}$$

Using the measured expired gas volume from the DB, V_E_ (ATPS, Atmospheric Temperature Pressure Saturated), which was then used to calculate V_E_ (BTPS) and V_E_ (STPD) by applying the appropriate correction factor.

### Experimental design

Prior to testing, participants had their body mass and height measured using a physician scale and stadiometer, respectively, in light clothing without shoes. Participants were then fitted with a mouthpiece, nose clip, and headgear that were used with the DB measurements and separately were fitted for the appropriate face mask to be used for the K5 testing. Participants then completed seated rest on a Lode Excalibur Sport (Groningen, The Netherlands) electronically braked cycle ergometer followed by pedaling at 50, 100, 150, 200, and 250 W. After completing the 150 W stage, participants were offered a 5–10 minute break before completing the final two stages. In instances where a participant could not complete 200 W or 250 W, they were asked to return on a second day (within a week) to complete those stages.

For each participant, the order of respiratory gas collection (DB, BxB, MC) was selected from the following three combinations to account for possible order effects and respiratory drift so each combination was completed by the same number of participants: 1) DB-MC-BxB (n = 5), 2) DB-BxB-MC (n = 5), or 3) MC-BxB-DB (n = 5). Three other combinations were possible (e.g. BxB-DB-MC), but were not included in the experimental design as during pilot testing they added a minimum of 5 minutes to each stage, resulting in an increase of more than 30 minutes to the whole protocol. The added time was due to extra switching of the masks/mouth piece as well as extra time for equilibration of the K5 MC system.

Table 1 shows the general timeline for testing during the resting condition and one cycling work rate (e.g. 50 W); the other cycling work rates followed the same timeline. During each stage the participant was connected to the DB or K5 (BxB or MC mode) for the first four minutes to reach steady state, followed by a 2-min gas collection period. The exception was that we used a 5-min gas collection at rest for DB and a 3-min gas collection at rest for K5. In rare cases at 250W, the DB was filled to capacity prior to the time ending, so the DB trial ended early. After switching to a different respiratory gas collection method, additional samples were collected for 2-min periods (except for a 5-min collection at rest for DB and a 3-min collection at rest for K5), and then this was repeated for the third respiratory gas collection method.

**Table 1:** Timeline for each testing combination for the resting condition and cycling work rates.

		Combination 1: DB/BxB/MC		Combination 2: DB/MC/BxB			Combination 3: MC/BxB/DB	
Condition	Time (minutes)	Method	Status	Method	Status	Time (minutes)	Method	Status
Rest	0–4	DB	Wash Out	DB	Wash Out	0–4	MC	Wash Out
	4–9	DB	Collection	DB	Collection	4–7	MC	Collection
	9–10	BxB	Mask Change	MC	Mask Change	7–8	BxB	System Change
	10–13	BxB	Collection	MC	Collection	8–11	BxB	Collection
	13–14	MC	System Change	BxB	System Change	11–12	DB	Mask Change
	14–17	MC	Collection	BxB	Collection	12–17	DB	Collection
Cycling Work Rate (e.g. 50 W)	0–4	DB	Steady State	DB	Steady State		MC	Steady State
	4–6	DB	Collection	DB	Collection		MC	Collection
	6–7	BxB	Mask Change	MC	Mask Change		BxB	System Change
	7–9	BxB	Collection	MC	Collection		BxB	Collection
	9–10	MC	System Change	BxB	System Change		DB	Mask Change
	10–12	MC	Collection	BxB	Collection		DB	Collection

DB, Douglas bag; MC, Cosmed K5 mixing chamber mode; and BxB, Cosmed K5 breath-by-breath mode. Note, for only the resting condition, due to differences in the DB collection time, combination 3 had a different time for each status point.

### Statistical analyses

Statistical analyses were carried out jointly using R and IBM SPSS statistical software version 25.0 (IBM, Armonk, NY). For all analyses, an alpha level of 0.05 was used to indicate statistical significance. The final two minutes of each stage (final three minutes at rest) from the K5 MC and BxB tests were averaged (60-s epochs) and compared with the DB collection for each stage. Two approaches were taken to examine the differences between the DB and K5 metabolic variables. First, repeated measures ANOVAs were used to compare metabolic variables (V_E_, VO_2_, VCO_2_, RER, F_E_O_2_, and F_E_CO_2_) measured by each system (DB, BxB, MC). Separate ANOVAs were performed for rest and each cycling work rate and metabolic variable. Pairwise comparisons with Bonferroni adjustments were performed to locate significant differences between devices, when necessary. Second, group level estimates for K5 MC and BxB modes were compared to DB using 95% equivalence testing with ±10% equivalence zones, as described by Dixon et al. [11]. Specifically, 90% confidence intervals were constructed for the paired (K5 minus DB) differences, and equivalence was defined as a confidence interval with upper and lower bounds that were each within ±10% of the DB mean. Separate tests were performed for each work rate and metabolic variable.

Additionally, paired t-tests were used to compare breathing frequency (Rf) and tidal volume (TV) measures between the K5 MC and BxB modes for rest and each cycling work rate. To examine individual variability, modified Bland-Altman plots were used to graphically show the variability in the individual error scores (DB minus K5 MC or BxB) over the complete range of measured values [12]. For examination of practical differences, we have defined a meaningful difference for accuracy (group level error) as greater than a 5% difference from DB values and precision (individual level error) as greater than a 10% difference from DB values. Using percentage difference rather than absolute differences reduce the concern that VO_2_ errors are generally larger at greater work rates. The 5% value is based on studies showing that the test-retest reliability in VO_2_ (using the exact same method) is usually greater than 0.85, and mean VO_2_ values are within 5% when comparing two different trials [13]. The 10% value is based on the fact that the minimum detectable change (MDC), expressed as a percent of measurement mean was less than 10% in a study of the Cosmed K4b^2^ versus DB. MDC indicates the magnitude of change needed to provide confidence that a change is not the result of random variation or measurement error [13].

## Results

Two participants could not complete cycling at 250W, six participants did not achieve metabolic steady state (1 at 200 W and 5 at 250W), and equipment malfunctions resulted in BxB data for one participant being removed at 150 W and DB values for one participant being removed at 100, 150, and 200 W. Thus, the final analytic sample used for analysis was: rest (n = 15), 50W (n = 14), 100W (n = 14), 150W (n = 13), 200W (n = 13), and 250W (n = 8). Physical characteristics (mean (SD)) of the participants were: age, 30.6 (7.4) yrs; height, 181.2 (6.5) cm; weight, 81.3 (16.7) kg; and BMI, 24.8 (5.1) kg^.^m^2^.

Table 2 shows the physiological responses measured by each respiratory gas collection method. In general, the results of the equivalence testing and ANOVA testing were similar for all variables except for the K5 MC V_E_, VCO_2_, and RER. The results below are presented based on the ANOVA testing.

**Table 2:** Physiological responses measured during rest and five work rates on a cycle ergometer using Douglas bags, and Cosmed K5 portable metabolic system mixing chamber and breath-by-breath modes.

	Douglas Bag	K5 Mixing Chamber	K5 Breath-by-Breath
**V**_**E**_ **(BTPS, l/min)**			
Rest (n = 15)	13.8 ± 2.4	13.3 ± 1.8^	13.0 ± 1.7^
50 W (n = 14)	31.1 ± 4.1	29.9 ± 5.0^	31.0 ± 4.8^#^
100 W (n = 14)	44.8 ± 5.9	43.2 ± 7.2	43.4 ± 7.0
150 W (n = 13)	57.5 ± 6.1	57.8 ± 5.2	57.4 ± 4.8
200 W (n = 13)	75.2 ± 13.2	76.7 ± 12.1	77.4 ± 12.8
250 W (n = 8)	96.0 ± 12.1	98.6 ± 16.9	96.9 ± 14.9
**VO**_**2**_ **(STPD, l/min)**			
Rest	0.38 ± 0.06	0.43 ± 0.06*^	0.41 ± 0.05^
50 W	1.16 ± 0.14	1.16 ± 0.18	1.20 ± 0.19
100 W	1.65 ± 0.14	1.64 ± 0.20	1.70 ± 0.17^#^
150 W	2.19 ± 0.14	2.17 ± 0.16	2.25 ± 0.14
200 W	2.77 ± 0.16	2.74 ± 0.21	2.85 ± 0.20
250 W	3.43 ± 0.23	3.35 ± 0.26	3.39 ± 0.30
**VCO**_**2**_ **(STPD, l/min)**			
Rest	0.33 ± 0.06	0.36 ± 0.05^	0.34 ± 0.05^
50 W	1.04 ± 0.13	1.01 ± 0.16^	1.05 ± 0.16
100 W	1.53 ± 0.16	1.44 ± 0.20^	1.47 ± 0.17
150 W	2.03 ± 0.15	1.93 ± 0.16	1.91 ± 0.12*
200 W	2.61 ± 0.19	2.49 ± 0.17	2.46 ± 0.16*
250 W	3.33 ± 0.31	3.09 ± 0.33^	3.02 ± 0.18*^
**F**_**E**_**O**_**2**_			
Rest	0.1742 ± 0.0051	0.1699 ± 0.0024*	0.1710 ± 0.0030
50 W	0.1625 ± 0.0023	0.1617 ± 0.0036	0.1618 ± 0.0040
100 W	0.1629 ± 0.0034	0.1626 ± 0.0035	0.1611 ± 0.0041^#^
150 W	0.1613 ± 0.0036	0.1631 ± 0.0043	0.1615 ± 0.0033
200 W	0.1625 ± 0.0058	0.1648 ± 0.0045*	0.1637 ± 0.0061
250 W	0.1642 ± 0.0051	0.1667 ± 0.0054	0.1656 ± 0.0068
**F**_**E**_**CO**_**2**_			
Rest	0.0308 ± 0.0042	0.0341 ± 0.0026*^	0.0342 ± 0.0046*^
50 W	0.0424 ± 0.0021	0.0431 ± 0.0026	0.0432 ± 0.0034
100 W	0.0432 ± 0.0031	0.0424 ± 0.0032	0.0433 ± 0.0036
150 W	0.0447 ± 0.0031	0.0424 ± 0.0035*	0.0425 ± 0.0030*
200 W	0.0444 ± 0.0048	0.0416 ± 0.0041*	0.0410 ± 0.0049*^
250 W	0.0440 ± 0.0036	0.0402 ± 0.0042*^	0.0402 ± 0.0060^
**RER**			
Rest	0.87 ± 0.05	0.83 ± 0.06	0.83 ± 0.6
50 W	0.90 ± 0.03	0.87 ± 0.04	0.88 ± 0.05
100 W	0.93 ± 0.04	0.88 ± 0.04*	0.86 ± 0.05*^#^
150 W	0.93 ± 0.04	0.89 ± 0.04*	0.85 ± 0.04*^#^
200 W	0.94 ± 0.04	0.91 ± 0.05*	0.87 ± 0.06*^#^^
250 W	0.97 ± 0.05	0.92 ± 0.04*	0.90 ± 0.05*^
**Rf (breaths/min)**			
Rest		18 ± 1	17 ± 1
50 W		22 ± 3	22 ± 3
100 W		26 ± 5	26 ± 5
150 W		30 ± 6	29 ± 6
200 W		33 ± 7	34 ± 9
250 W		37 ± 6	37 ± 8
**TV (BTPS, l)**			
Rest		0.8 ± 0.2	0.8 ± 0.1
50 W		1.4 ± 0.2	1.4 ± 0.2
100 W		1.7 ± 0.3	1.7 ± 0.3
150 W		2.0 ± 0.3	2.0 ± 0.3
200 W		2.4 ± 0.3	2.4 ± 0.3
250 W		2.7 ± 0.3	2.7 ± 0.4

Values are means ± SD. BTPS, body temperature pressure saturated; STPD, standard temperature pressure dry; V_E_, minute ventilation; VO_2_, oxygen uptake; VCO_2_, carbon dioxide production; F_E_O_2_, fraction of oxygen in expired air; F_E_CO_2_, fraction of carbon dioxide in expired air; RER, respiratory exchange ratio; Rf, respiratory rate; TV, tidal volume.

*significantly different from the Douglas bag

^#^significantly different from the Cosmed K5 mixing chamber mode

^not significantly equivalent with the Douglas bag.

The K5 MC mode was not statistically significantly different from DB at rest or any cycling work rate for V_E_ or VCO_2_ (all, p≥0.05). For VO_2_, the K5 MC mode was not significantly different from the DB mode at any cycling work rate (all, p≥0.05); however, it significantly overestimated DB VO_2_ at rest by 0.05 l/min (p = 0.006). The K5 MC mode was significantly different from DB F_E_O_2_ at rest (mean difference (DB-K5 MC); +0.0043) and 200W (-0.0023) and DB F_E_CO_2_ at rest (-0.0033), 150W (-0.0023), 200W (+0.0028), and 250W (+0.0038). In addition, the K5 MC mode significantly underestimated DB RER by 0.03 to 0.05 at 100W, 150W, 200W, and 250W (all, p<0.05).

The K5 BxB mode was not significantly different from DB at rest or any work rate for V_E_, VO_2_, or F_E_O_2_ (p≥0.05). For VCO_2_, the K5 BxB mode significantly underestimated DB VCO_2_ at 150W, 200W, and 250W by 0.12, 0.14, and 0.31 l/min, respectively (all, p<0.05). The K5 BxB was also significantly different from DB F_E_CO_2_ at rest (mean difference (DB-K5 BxB); -0.0034), 150W (+0.0022), and 200W (+0.0034), and significantly underestimated DB RER by 0.06 to 0.08 at 100W, 150W, 200W, and 250W (all, p<0.05).

There were no significant differences between the K5 MC and BxB modes at rest or any work rate for VCO_2_, F_E_CO_2_, Rf, or TV (all, p≥0.05). The K5 MC was significantly lower than K5 BxB for V_E_ at 50W by -1.1 l/min and VO_2_ at 100W by 0.07 l/min (all, p<0.05). The K5 MC was significantly higher than the K5 BxB mode for measurement of F_E_O_2_ at 100W by 0.0015, and RER at 100W, 150W, and 200W by 0.01 to 0.04 (all, p<0.05).

**Fig 1:**
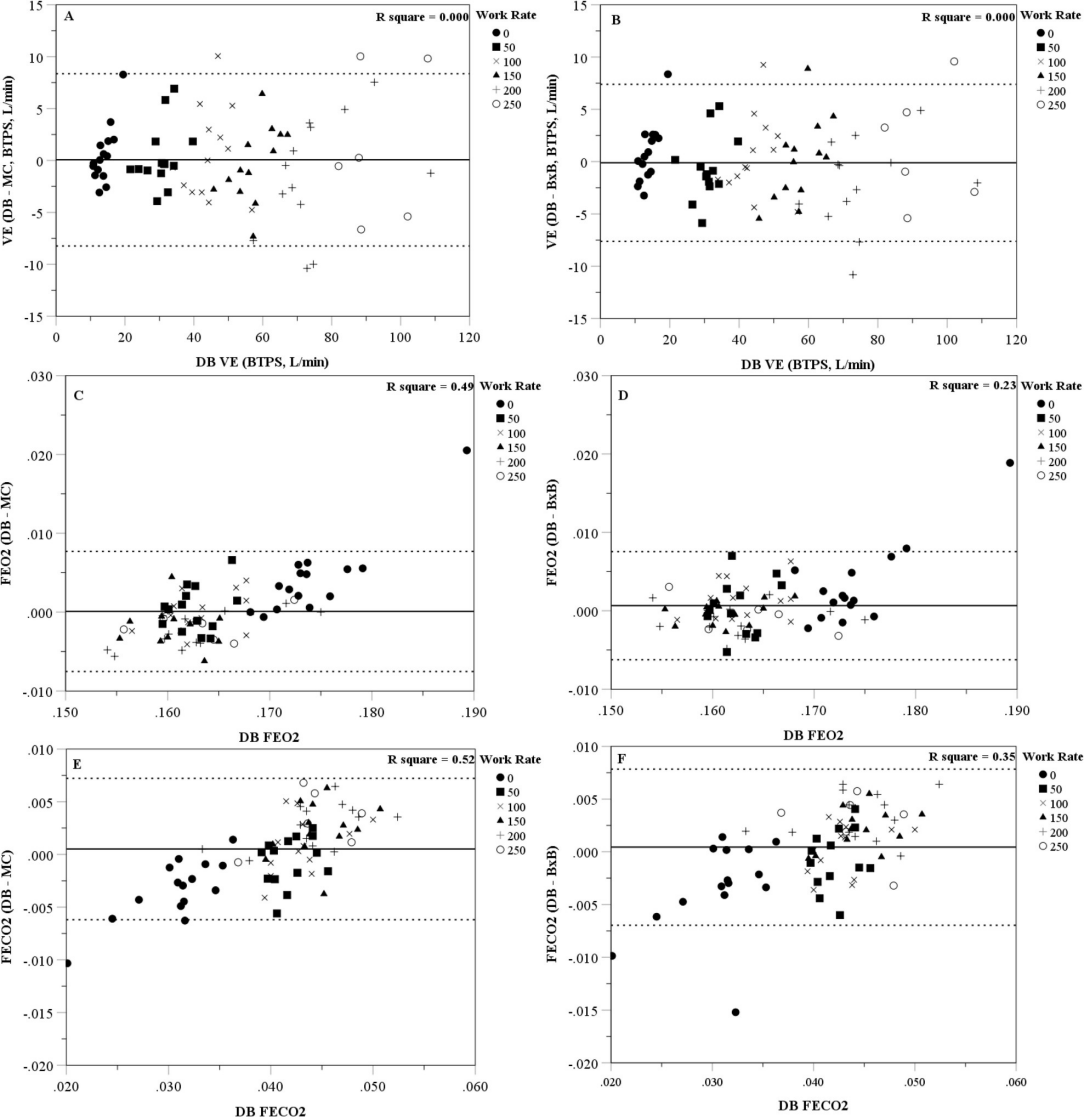
Bland-Altman plots of the error scores (Douglas bag (DB) minus computerized system) for: A) Cosmed K5 mixing chamber (MC) minute ventilation (V_E_), B) Cosmed K5 breath-by-breath (BxB) V_E_, C) MC fraction of expired oxygen (F_E_O_2_), D) BxB F_E_O_2_, E) MC fraction of expired carbon dioxide (F_E_CO_2_), and F) BxB F_E_CO_2_. Solid line represents the mean difference; dashed line represents the 95% limits of agreement.

**Fig 2:**
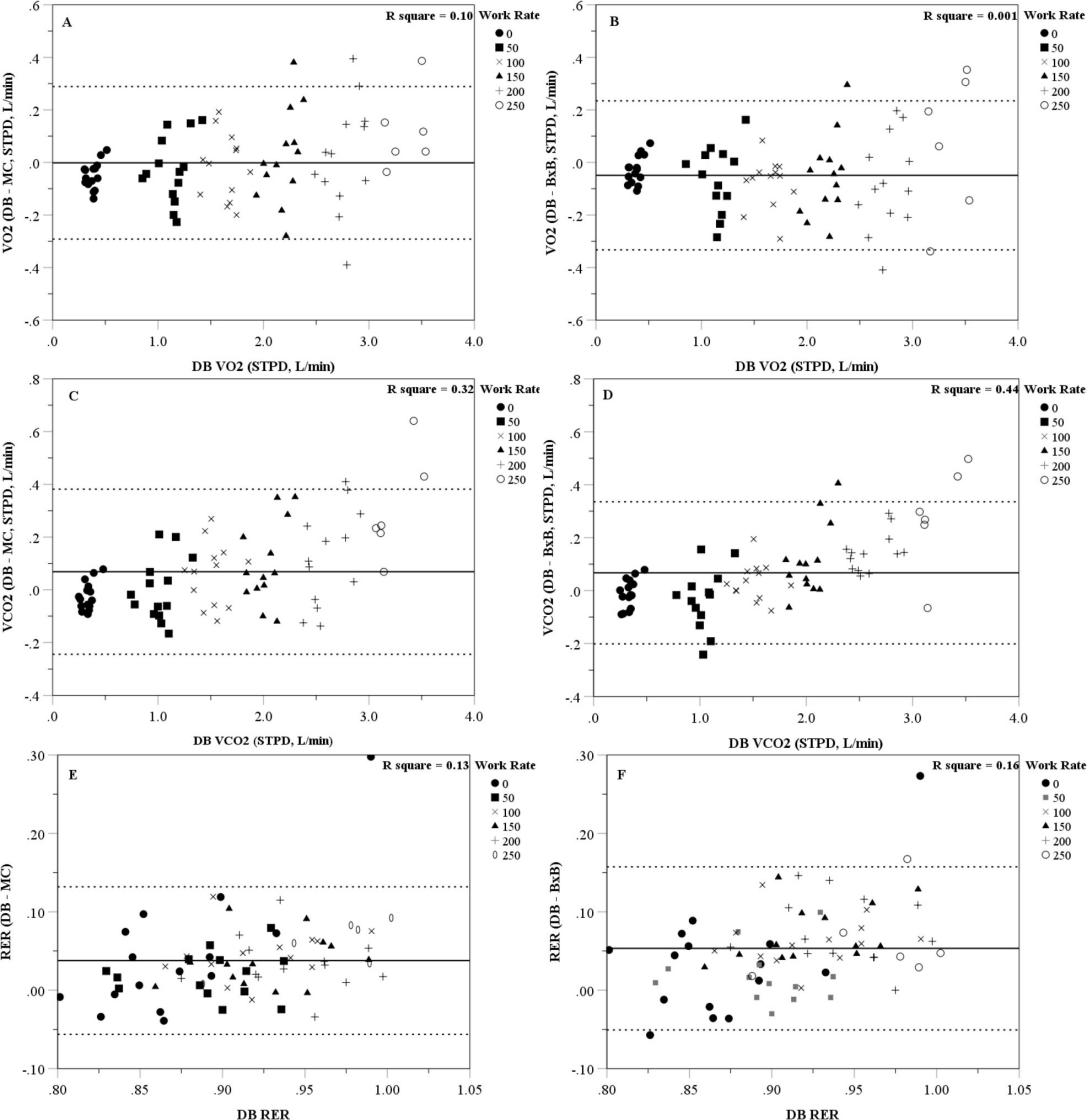
Bland-Altman plots of the error scores (Douglas bag minus computerized system) for oxygen consumption (VO_2_) and carbon dioxide production (VCO_2_). A) Cosmed K5 mixing chamber (MC) oxygen consumption (VO_2_), B) Cosmed K5 breath-by-breath (BxB) VO_2_, C) MC and carbon dioxide production (VCO_2_), D) BxB VCO_2_, E) MC respiratory exchange ratio (RER), and F) BxB RER. Solid line represents the mean difference; dashed line represents the 95% limits of agreement.

Figs (1A–1F) and (2A–2F) are Bland-Altman plots showing the individual difference scores (DB minus K5 MC or BxB mode) for each physiological variable measured. Table 3 shows the mean bias, lower and upper 95% prediction interval, and the percent of participants that were within 10% of the DB value for each variable and work rate. Overall, when rest and all work rates are examined together, there was close agreement at the group level and acceptable limits of agreement between the DB method and the K5 MC and BxB modes for most metabolic variables; however, the K5 MC and BxB modes tended to slightly overestimate FECO2 at lower work rates and slightly underestimate FECO2 at higher work rates. When examining the metabolic variables at each work rate separately, the resting measures were the least precise with less than half of the participants having K5 MC or BxB values within 10% of the DB VE, VO2 and VCO2 values. However, precision improved during exercise and between 100 and 250W the majority of participants had K5 MC and BxB values within 10% of the DB values (across all variables examined).

**Table 3:** Mean bias [Douglas bag (DB) minus K5], lower (LL) and upper (UL) 95% prediction interval, and number (n) of participants that were within 10% of the measured DB value.

	K5 Mixing Chamber		K5 Breath-by-Breath	
	Mean Bias (LL, UL)	n≤10% of DB (%)	Mean Bias (LL, UL)	n≤10% of DB (%)
**V**_**E**_ **(BTPS, L/min)**				
Rest (n = 15)	0.54 (-4.95, 3.34)	6 (40.0)	0.79 (-4.78, 3.63)	6 (40.0)
50 W (n = 14)	0.29 (-5.58, 3.28)	10 (71.4)	-0.65 (-6.77, 2.48)	9 (64.3)
100 W (n = 14)	0.7 (-7.9, 5.09)	10 (71.4)	0.48 (-6.99, 4.29)	11 (78.6)
150 W (n = 13)	-0.34 (-7.45, 3.28)	11 (84.6)	0.12 (-7.71, 4.11)	11 (84.6)
200 W (n = 13)	-1.51 (-12.53, 4.11)	10 (76.9)	-2.14 (-10.53, 2.14)	11 (84.6)
250 W (n = 8)	1.25 (-12.91, 8.48)	5 (62.5)	1.38 (-9.42, 6.89)	8 (100.0)
**VO**_**2**_ **(STPD, L/min)**				
Rest	-0.05 (-0.15, 0)	7 (46.7)	-0.03 (-0.13, 0.02)	6 (40.0)
50 W	-0.03 (-0.27, 0.1)	6 (42.9)	-0.06 (-0.31, 0.06)	7 (50.0)
100 W	-0.02 (-0.27, 0.11)	9 (64.3)	-0.08 (-0.27, 0.02)	11 (78.6)
150 W	0.02 (-0.33, 0.2)	11 (84.6)	-0.05 (-0.36, 0.1)	10 (76.9)
200 W	0.02 (-0.39, 0.23)	11 (84.6)	-0.08 (-0.43, 0.1)	11 (84.6)
250 W	0.12 (-0.17, 0.26)	7 (87.5)	0.07 (-0.46, 0.34)	6 (75.0)
**VCO**_**2**_ **(STPD, L/min)**				
Rest	-0.02 (-0.13, 0.03)	3 (20.0)	-0.01 (-0.12, 0.05)	7 (46.7)
50 W	0 (-0.23, 0.12)	9 (64.3)	-0.03 (-0.26, 0.08)	8 (57.1)
100 W	0.06 (-0.18, 0.18)	11 (78.6)	0.03 (-0.1, 0.1)	12 (85.7)
150 W	0.1 (-0.21, 0.26)	9 (69.2)	0.11 (-0.15, 0.25)	10 (76.9)
200 W	0.12 (-0.24, 0.3)	11 (84.6)	0.14 (0, 0.22)	12 (82.3)
250 W	0.31 (-0.09, 0.51)	4 (50.0)	0.28 (-0.1, 0.48)	5 (62.5)
**F**_**E**_**O**_**2**_				
Rest	0.0043 (-0.0057, 0.0093)	14 (93.3)	0.0032 (-0.0072, 0.0085)	15 (100.0)
50 W	0.0004 (-0.0053, 0.0033)	14 (100.0)	0.0004 (-0.0065, 0.0039)	14 (100.0)
100 W	0.0001 (-0.0047, 0.0025)	14 (100.0)	0.0015 (-0.0034, 0.0039)	14 (100.0)
150 W	-0.0018 (-0.0068, 0.0007)	13 (100.0)	-0.0002 (-0.0032, 0.0012)	13 (100.0)
200 W	-0.0024 (-0.0067, -0.0001)	13 (100.0)	-0.0012 (-0.0051, 0.0008)	13 (100.0)
250 W	-0.0017 (-0.0057, 0.0003)	8 (100.0)	-0.0009 (-0.0054, 0.0014)	8 (100.0)
**F**_**E**_**CO**_**2**_				
Rest	-0.0033 (-0.009, -0.0004)	9 (60.0)	-0.0034 (-0.0121, 0.001)	9 (60.0)
50 W	-0.0006 (-0.0052, 0.0017)	13 (92.9)	-0.0007 (-0.0063, 0.0021)	12 (85.7)
100 W	0.0008 (-0.0045, 0.0036)	11 (78.6)	0.0003 (-0.0045, 0.0027)	14 (100.0)
150 W	0.0023 (-0.0029, 0.0049)	10 (76.9)	0.0023 (-0.0016, 0.0042)	11 (84.6)
200 W	0.0028 (-0.0014, 0.0049)	10 (76.9)	0.0034 (-0.0011, 0.0056)	18 (61.5)
250 W	0.0033 (-0.0022, 0.0061)	5 (62.5)	0.0027 (-0.0035, 0.0058)	4 (50.0)
**RER**				
Rest	0.05 (-0.12, 0.13)	12 (80.0)	0.04 (-0.12, 0.12)	13 (86.7)
50 W	0.02 (-0.04, 0.05)	13 (92.9)	0.02 (-0.05, 0.05)	13 (92.9)
100 W	0.05 (-0.02, 0.08)	13 (92.9)	0.06 (0, 0.09)	12 (85.7)
150 W	0.04 (-0.03, 0.07)	12 (92.3)	0.07 (0, 0.11)	9 (69.2)
200 W	0.03 (-0.04, 0.07)	12 (92.3)	0.08 (-0.01, 0.12)	8 (61.5)
250 W	0.06 (0, 0.09)	8 (100.0)	0.06 (-0.04, 0.12)	6 (75.0)

BTPS, body temperature pressure saturated; STPD, standard temperature pressure dry; V_E_, minute ventilation; VO_2_, oxygen uptake; VCO_2_, carbon dioxide production; F_E_O_2_, fraction of oxygen in expired air; F_E_CO_2_, fraction of carbon dioxide in expired air; RER, respiratory exchange ratio.

## Discussion

The purpose of this study was to test the accuracy of the Cosmed K5 MC and BxB modes against the criterion DB method. A primary finding of this study was that V_E_ and VO_2_ values from the K5 MC and BxB modes were not significantly different from the criterion DB values at any cycling work rate. For VO_2_, the values from the DB and both K5 modes were within 0.08 l/min at rest and all cycling work rates. The errors seen in the current study are similar to that of the K5’s predecessor (K4b^2^) that had mean errors for VO_2_ of less than 0.1 l/min, compared to DB [3].

VCO_2_ values from the K5 MC and BxB modes were not different from DB values up to 100W, but tended to be lower than DB values at 150W, 200W, and 250W by 0.10 to 0.31 l/min. However, only the K5 BxB VCO_2_ values were significantly lower than the DB values at ≥150W. This is likely due to the lower F_E_CO_2_ values for both K5 modes at those intensities since the V_E_ values were not significantly different between either K5 mode and DB. This is in contrast to a K4b^2^ validation study that also showed lower VCO_2_ values at higher intensities, but the major contributing factor in that study was a significantly lower V_E,_ compared to DB, since F_E_CO_2_ was not different at the same intensities [3].

For both K5 modes, RER values at all cycling intensities were lower than the DB method, due to the K5 generally providing lower VCO_2_ values than the DB method. This is similar to the results of a previous K4b^2^ validation study in which RER was significantly underestimated at every intensity. In a study by McLaughlin et al. [3], the RER underestimations up to 200W appeared to be due to an overestimation of VO_2_, while at 200W and 250W, the lower VCO_2_ was the contributing factor. It appears that the new K5 VO_2_ measurements have been improved across all intensities; however, the K5 VCO_2_ measurements are still underestimated at higher work rates.

In terms of practical differences seen during the testing, similar trends were seen in both the group and individual level errors for VE, VO2, and VCO2. The mean group level errors, compared to the DB, for were greatest during rest for both the K5 MC (4.0% for VE and 11.6% for VO2) and K5 BxB (6.1% for VE and 7.0% for VO2); however, the mean errors were less than 5% during all cycling work rates. In contrast, for VCO2, the highest mean group level errors were seen at 250W for the K5 MC and BxB modes (9.9% and 9.3%, respectively). For precision (individual level error), K5 MC and BxB VE, VO2, and VCO2, all had the worst precision at rest with less than half the participants having values within 10% of the DB values. The K5 MC and BxB modes were most precise for measurement of VE, VO2, and VCO2 during cycling work rates between 100W and 200W where the majority of participants had K5 values within 10% of DB values.

For the comparisons between the K5 MC and BxB modes, both modes provided similar physiological measures for rest and across all cycling intensities. Where there were statistically significant differences, they did not represent meaningful differences from a practical standpoint. For example, the V_E_ (50W) and VO_2_ (150W) measures were different by only l.1 l/min (3.6%) and 0.07 l/min (3.5%), respectively. This is important and suggests that researchers can be confident in values between the two different modes being comparable to each other, as well as with the criterion DB method.

The Cosmed MC and BxB modes each have their advantages and disadvantages. In theory, the MC should provide more stable measurements during steady-state testing, while BxB should have a greater ability to track rapid fluctuations in respiratory gas exchange variables with the onset and cessation of exercise, due to the instantaneous nature of the measurements. Thus, researchers should pick the best mode for their research design (the reader is referred to Ward [14] for more detailed information on the use of MC and BxB in testing). One important note on the K5 MC mode is that there is a washout period of up to 5 minutes before data collection can begin. This is due to the gas being sampled in direct proportion to the Rf and it takes time to wash out the room air from the MC within the K5 unit. In general, this washout period is longest at rest and occurs more quickly (within a couple minutes) at higher intensities. This is an important consideration for testing where a participant is wearing the K5 for extended periods of time and may need to remove the mask for bathroom or water breaks. When the mask is removed, the MC will begin sampling room air. Thus, when the participant replaces the mask to start testing again there will be a delay before data collection starts again due to the MC being washed out. In these types of testing protocols, the BxB mode is a better option as testing can resume immediately. Additionally, for studying O_2_ uptake kinetics, the BxB mode responds more quickly and is better able to track changes in VO_2_.

The current study is not without limitations. The sample was composed of primarily males (only one female) with high levels of cardiorespiratory fitness. Even with a fit group of participants, not all of them were able to complete the last 1–2 stages. However, the oxygen cost of cycling is consistent across populations regardless of fitness status [15]. Only seated rest and cycling were examined in the current study, thus it is not clear how valid the K5 is for other activities. However, a wide range of intensities were included in the current study providing confidence in values obtained during steady-state activity. The classical DB technique is traditionally performed using the micro-Scholander method [16]. Using electronic gas analyzers as we did, while common practice with the DB method now, could introduce error into the gas fraction measurements. Lastly, while the measurements for each system were made on the same day, they were not made simultaneously. Thus, some error could be introduced due to drifts in ventilation and oxygen consumption during each stage. However, the use of trained participants with cycling experience should reduce potential drift.

## Conclusions

The findings from the current study suggest that the K5 MC and BxB modes are both acceptable for the measurement of V_E_ and VO_2_ across a wide range of exercise intensities. Any differences from the criterion DB values were minimal, and are not considered to be of practical significance for most applications. Caution should be used for resting measures as both group and individual errors were statistically and meaningfully different. Lastly, when choosing a K5 mode to use during testing, researchers can be confident that both the K5 MC and BxB modes provide similar values to each other. There were no significant differences in VCO_2_ for seated rest up to 100 W, but at higher work rates the Cosmed K5 BxB mode showed a slight underestimation of VCO_2_. Additionally, at higher work rates both Cosmed modes significantly underestimated RER, which could affect measurement of substrate utilization. Further testing is warranted to assess the accuracy of the K5 MC and BxB modes during different modes of exercise and various environmental conditions.

## Supporting information

S1 File (XLSX)
